# Sella Turcica Morphology in Patients With Genetic Syndromes: Protocol for a Systematic Review

**DOI:** 10.2196/16633

**Published:** 2020-11-05

**Authors:** Imaan Amina Roomaney, Manogari Chetty

**Affiliations:** 1 Department of Oral Biology and Dental Genetics Faculty of Dentistry University of Western Cape Cape Town South Africa

**Keywords:** sella turcica, craniofacial syndrome, craniofacial malformations, cephalometrics, systematic review

## Abstract

**Background:**

The sella turcica is an important anatomical reference used in orthodontics and the evaluation of craniofacial growth. Studies have found an association between variations in sella turcica morphology in patients with certain syndromes affecting the craniofacial complex. It is hypothesized that each related syndrome or pathological condition is associated with a specific pattern of malformation of the sella turcica.

**Objective:**

This study outlines the protocol for a systematic review that aims to determine if genetic syndromes involving the craniofacial complex are associated with abnormal radiographic sella turcica morphology and if there is a pattern of malformation that is consistent with each syndrome.

**Methods:**

An electronic database search was conducted using a planned search strategy to identify relevant studies. We included primary studies evaluating the morphology of the sella turcica based on imaging from a lateral view. Specifically, only studies with postnatal human participants with genetic syndromes involving the craniofacial complex were included in this review. We placed no restrictions on the language or time frame of these studies. Based on the search findings, studies were further screened for relevance and eligibility by two independent reviewers. Data were extracted from the selected studies. We assessed the selected studies for risk of bias and quality by using risk of bias tools from the Joanna Briggs Institute. We will provide a narrative synthesis of our findings and a structured summary based on prespecified themes.

**Results:**

The protocol is registered with PROSPERO (#CRD42019148060) and approved by the University of Western Cape Biomedical Science Research Ethics Committee (BM205/3). The literature search was conducted in September 2019 and updated in July 2020. The study was completed in August 2020, and the findings will be published in an open-access journal.

**Conclusions:**

The results of this systematic review are expected to provide a comprehensive list of morphological variations of the sella turcica, which will aid in the identification of syndromes associated with the craniofacial complex. We also expect to identify patterns of sella turcica morphology that highlight genotype-phenotype correlations, thus adding to the body of evidence relating to genetics and craniofacial malformations.

**Trial Registration:**

PROSPERO International Prospective Register of Systematic Reviews CRD42019148060; https://www.crd.york.ac.uk/prospero/display_record.php?RecordID=148060

**International Registered Report Identifier (IRRID):**

RR1-10.2196/16633

## Introduction

The sella turcica is an important anatomical reference used in orthodontics and the evaluation of craniofacial growth [1]. It is easily determinable on the lateral profile and cephalometric radiographs and is useful in analyzing the relationship of the maxilla and mandible to the cranium, and to each other [2]. The sella turcica has several morphological variations that can be examined radiographically. Studies have found an association between variations in the sella turcica morphology in patients with dental anomalies [3-5] and those with certain syndromes affecting the craniofacial complex [6-9].

The sella turcica is an important region for the migration of neural crest cells to the frontonasal and maxillary developmental fields during embryological development [10,11]. The pituitary gland is located within the pituitary fossa, which is situated in the body of the sphenoid bone, within the cranium [10,12]. The pituitary gland is particularly important for endocrine function, and the development of the sella turcica is closely associated with that of the pituitary gland [13,14]. Thus, anomalies in the pituitary gland often present as abnormalities in the sella turcica [2,15]. Pathology of the pituitary gland often presents as aberrations in the size of the sella turcica. Pathological conditions such as adenomas, Rathke cleft cysts, and aneurysms are associated with enlargement of the sella turcica [14]. In contrast, hypopituitarism, growth hormone deficiency, and necrosis of the pituitary gland may result in a smaller sella turcica [14]. Moreover, numerous studies have found an association between conditions and syndromes affecting the craniofacial complex and morphological deviations of the sella turcica. These include Williams syndrome [8], Down syndrome [6], osteogenesis imperfecta [16], Axenfeld-Rieger syndrome [9], cleft-lip and palate [7], trisomy 18, Fragile X syndrome, and Cri-du-chat syndrome [1].

Several classifications describe the morphological variations of the sella turcica based on radiographic findings. Its morphology is often described subjectively and qualitatively, and it is classified into shapes such as circular, oval, flat, shallow, and J-shaped [17]. Several studies have examined the prevalence and distribution of different morphological presentations of the sella turcica in “normal” individuals to produce reference standards [2,15,17-19]. These reference standards assist in detecting deviations from the norm and pathology in the sella turcica [2].

According to Kjaer et al [1], each syndrome or pathological condition has a specific pattern of malformation of the sella turcica, which varies in severity depending on the phenotype. Therefore, this systematic review aimed to determine whether genetic syndromes involving the craniofacial complex are associated with abnormal sella turcica morphology and to identify patterns of malformations if they exist. The study also aimed to explore the clinical relevance of these findings.

## Methods

### Study and Ethics Approvals

This protocol has been compiled using the PRISMA-P (Preferred Reporting Items for Systematic reviews and Meta-Analyses for Protocols) guidelines [20] and is registered with PROSPERO (#CRD42019148060). The systematic review conformed to the PRISMA guidelines [21].

Ethics approval and consent for participation were not required for this study. This study was approved by the University of Western Cape Biomedical Science Research Ethics Committee (BM205/3).

### Study Eligibility Criteria

The literature search included primary studies evaluating the morphology of the sella turcica based on lateral radiographs or via digital imaging from a lateral view, such as cone-beam computed tomography (CBCT). Only studies on postnatal human participants with genetic syndromes primarily involving the craniofacial complex were included in the review. Reviews and letters to the editor were excluded. No restriction was placed on grey literature, language, and time frame. The inclusion criteria were deliberately left broad to attempt to find as many relevant papers that relate to the primary and secondary objectives of this study as possible, as the morphology of the sella turcica may be noted as an incidental finding in some published studies.

### Anatomic Structure of Interest

The sella turcica is a saddle-like bony structure on the upper surface of the sphenoid bone. The most posterior part of the sella turcica is known as the hypophyseal or pituitary fossa that houses the pituitary gland [22]. It has an anterior and a posterior border, known as the tuberculum sellae and the dorsum sellae, respectively [14]. The dorsum sellae is continuous with the clivus and the posterior clinoid process. The two anterior and posterior clinoid processes project over the pituitary fossa [10,14].

As noted in the introduction, the morphology of the sella turcica is often described subjectively and qualitatively as shapes that broadly describe the shape of the sella turcica itself and its relation to the tuberculum and dorsum sellae [17]. Axelsson et al [18] created a detailed classification consisting of six morphological types of the sella turcica: (a) “normal,” (b) oblique anterior wall, (c) double contour of the floor, (d) sella turcica bridge, (e) irregularity (notching) in the posterior wall, and (f) pyramidal shape of the dorsum sellae. Andredaki et al [17] established the need for more quantitative methods to measure the size and shape of the sella turcica by using specialized software. In the review, we considered any method used to describe the morphology of the sella turcica.

### Population of Interest

A syndrome is defined as a set of symptoms occurring together or a pattern of multiple malformations thought to be pathogenetically related and not known to represent a single sequence or polytopic field defect [23]. According to Kjaer [24], a craniofacial syndrome is characterized by morphological and developmental deviations in the cranial tissue components, including the teeth. Humans with genetic syndromes primarily involving the craniofacial bony complex were included in this study. However, primary endocrine syndromes or syndromes caused by neoplasia as well as empty sella syndrome were excluded.

### Imaging of the Sella Turcica

Imaging of the sella turcica for orthodontic and craniofacial growth assessment purposes is conventionally performed using either digital or nondigital lateral cephalometric radiographs. For this review, we also included studies involving lateral skull radiography and CBCT providing a lateral view of the sella turcica region [25]. Moreover, we also included studies providing an adequate description of the lateral view of the sella turcica with novel forms of imaging, given that measurements are correlated to a standard cephalometric radiograph.

### Study Outcomes

The primary objective was to determine if genetic syndromes involving the craniofacial complex are associated with an abnormal morphology of the sella turcica based on radiographic analysis. Thus, we needed to first establish what is considered a “normal” morphology of the sella turcica. Axelsson et al [18] described the U-shaped morphology of the sella turcica as normal based on a subjective, visual assessment. Andredaki et al [17] used a software program to determine normative reference standards of the sella turcica. Therefore, we allowed flexibility in the definition of “normal” morphology as there is no accepted standard at present. If an adequate description of the “normal” reference morphology were provided in the included studies, it would be reported. Deviation from the “normal” was defined as “abnormal.”

The secondary outcome measures relate to the findings of morphological aberrations of the sella turcica in individuals with the same syndrome as well as between them and individuals with no genetic syndromes if a comparator was used in the given study. This was done in the same manner as determining the primary outcome measure and then observing whether trends exist within and between studies.

### Information Source and Search Strategy

An electronic search of several databases and journals was conducted using a planned search strategy (Table 1). SCOPUS, PubMed/MEDLINE, Web of Science, ProQuest, and WorldCat, as well as all databases within EBSCOhost, were searched. Each electronic database was searched using a tailored keyword or MeSH term. The results of the search were documented, reported, and compared between databases. Where papers were found to be relevant, the reference lists were manually searched for additional relevant papers. Additional searches were conducted using the search term “sella turcica genetic syndrome” with Google Scholar, ISI Citation Indices, and Web of Science ISI proceedings (conference proceedings).

Unpublished and ongoing studies were also sought from online registries, including the NIH Health Services Research Projects in Progress, the ISRCTN registry, the UK Clinical Trials Gateway, and the WHO International Clinical Trials Platform. In cases wherein studies were found to be potentially relevant and full-text papers could not be found, the authors were contacted to gain more information and determine whether the study should be included.

**Table 1:** Example of search strategy used in this review (Database: PubMed; Date: Sep 24, 2019; Limits: none).

No.	Search terms	Result
1	SELLA[ALL FIELDS]	8384
2	SELLA TURCICA OR HYPOPHYSEAL FOSSA OR PITUITARY FOSSA[ALL FIELDS]	8384
3	#1 OR #2	8384
4	SYNDROME[Title/Abstract]	8,65,737
5	GENETIC SYNDROME[ALL FIELDS]	1,08,333
6	#4 AND #5	8,92,138
7	#3 AND #6	1202

### Study Selection

Papers were screened in two stages—initially by title and abstract screening and then by full-text screening. Two reviewers conducted the screening and data extraction independently, and any failure of consensus was resolved by a senior third party. Reasons for exclusion of studies will be provided in the report. Interrater agreement was calculated and reported.

### Data Extraction and Management

Each reviewer independently performed data extraction. An electronic data extraction form was custom-made, piloted, and amended as required for this review. The data collection form included the fields enlisted in Textbox 1.

**Data extraction fields used in this study.**

Study Information

- Authors, title, year of publication, journal
- Study designs
- County of study

Study Participants

- Recruitment procedures
- Baseline characteristics, demographics
- Type of syndrome
- Genetic information
- Any additional abnormalities or medical conditions
- Any therapies that the patients might have received (eg, bisphosphonates)

Methods

- Study design, study duration
- Allocation sequence concealment, blinding, other concerns about bias

Imaging Techniques

- Type of imaging used (cone-beam computed tomography, digital cephalogram, or other)
- Additional information regarding standardization of images

Outcomes

- Morphology of the sella turcica
- Method of measurement
- Method of classification
- Method of bias elimination in the determination of morphology

Results

- Determining the “normal” sella turcica within populations
- Prevalence of sella turcica abnormalities in patients with syndromes affecting the craniofacial complex
- Patterns of sella turcica morphology amongst the population
- Description of findings regarding sella turcica morphology

### Availability of Data and Materials

All data generated or analyzed during this study will be included in the published study.

### Study Quality and Risk of Bias Assessment

Each reviewer independently conducted an assessment of the study quality and risk of bias by using the risk of bias tools recommended by the Joanna Briggs Institute, for each included case study, case series, case-control study, and cohort study. Interrater agreement was then calculated. These assessments will be summarized and included in the report.

### Analysis of Study Findings

We will provide a narrative synthesis of the findings from the included studies. The narrative synthesis will be structured according to themes in the following manner, to provide a coherent summary of findings: characteristics of included studies; baseline characteristics of the population (eg, age and sex); nature of the syndrome (a brief overview of the syndromes included with a summary of craniofacial manifestations); any genetic information considered pertinent; imaging techniques used; techniques used to describe sella turcica morphology; methods of classification of sella turcica morphology; prevalence of sella turcica anomalies; patterns of sella turcica abnormalities within and between syndromes; and any additional findings. Limitations and gaps in the reporting and methodologies will be noted and discussed.

## Results

No patients were involved in the development of this study. This protocol was completed in September 2019 and registered with PROSPERO (#CRD42019148060) in December 2019. The electronic literature search was conducted in September 2019 and updated in July 2020. The original search yielded 11,844 titles. This study was completed in August 2020 and will be published soon. We intend to publish this study in an open-access journal to allow for the wide dissemination of findings and promote ease of access to the public.

## Discussion

### Principal Findings

This study aimed to determine if genetic syndromes involving the craniofacial complex are associated with abnormal sella turcica morphology and to identify patterns of malformations if they exist. Current evidence suggests that there is an association between morphological abnormalities of the sella turcica and abnormalities in craniofacial development and that these abnormalities appear to be patterned among patients with particular syndromes [1]. However, no systematic review has been conducted to confirm this.

The sella turcica is considered a borderline area between developmental fields [26]. The anterior part of the sella turcica is derived from the neural crest cells, whereas most of its posterior wall and floor is derived from the notochord mesoderm [27]. Thus, abnormalities in the anterior part of the sella turcica may be associated with malformations in the facial bones, and abnormalities in the posterior wall and floor may be associated with abnormalities in the spine [27]. The sella turcica bridge between the anterior and posterior walls is also more commonly observed in individuals with severe malocclusions and other craniofacial malformations [5,28]. Owing to this complex development and the close association between the central nervous system, peripheral nervous system, and endocrine system, malformation of the sella turcica may be affected by many genetic and nongenetic factors. However, the patterning of malformations within certain genotypes indicates that genes play a central role in these malformations. Thus, attempting to understand these malformation patterns may provide us with new avenues of genetic and craniofacial pathology research.

### Conclusion

The results of this systematic review are expected to provide a comprehensive list of morphological variations of the sella turcica, which will aid in the identification of syndromes associated with the craniofacial complex. It may also provide insights into patterns of craniofacial malformations that may be genetically linked, thus adding to the body of evidence relating to genetics and craniofacial malformations.

## Abbreviations

CBCT: cone-beam computed tomography
PRISMA-P: Preferred Reporting Items for Systematic reviews and Meta-Analyses for Protocols
